# Are Currently Selected Laboratory Animals Useful in the Research of How Female Hormones Influence Orthodontic Biomechanics?

**DOI:** 10.3390/ani13040629

**Published:** 2023-02-10

**Authors:** Małgorzata Peruga, Beata Kawala, Michał Sarul, Jakub Kotowicz, Joanna Lis

**Affiliations:** 1 Independent Researcher, 93-410 Łódź, Poland; 2 Department of Dentofacial Orthopaedics and Orthodontics, Wroclaw Medical University, 50-376 Wrocław, Poland; 3 Department of Integrated Dentistry, Wroclaw Medical University, Krakowska 26, 50-425 Wroclaw, Poland; 4 Independent Researcher, 37-700 Przemyśl, Poland; 5 Independent Adult Orthodontics Clinic, Department of Dentofacial Orthopaedics and Orthodontics, Wroclaw Medical University, 50-376 Wrocław, Poland

**Keywords:** veterinary ethics, qualitative and quantitative research designs, animal experimental model, steroid hormones

## Abstract

**Simple Summary:**

Animal experiments should be carried out in consultation with veterinarians, who, having clinical knowledge, will help doctors choose the appropriate animal model. Our review of the literature shows how the harmful and unethical duplication of research from other research centers can be. Familiarization with the experimental protocols on an animal model is important each time we want to later extrapolate the obtained results to the target species, i.e., human. This is due to the law on the implementation of the procedures on people. This article focuses on orthodontic teeth movement. While experiments with laboratory animals seem easy, there are many pitfalls. Our goal in this article is to collect data on the maintenance of laboratory animals as models and to critically analyze them based on our literature review.

**Abstract:**

Animal testing was and remains the only method of introducing a certain treatment and medical procedure on humans. On the other hand, animals have their rights resulting from applicable legal acts, including Directive 2010/63/EU and, indirectly, the World Medical Association International Code of Medical Ethics (Helsinki Declaration, 1975, amended 2000). Thus, the question arises whether the credibility of the results of hormonal and orthodontic tests obtained so far and their usefulness for the human population is scientifically justified and worth sacrificing laboratory animals for. Especially that, according to statistical data, about 50% of laboratory animals are euthanized at the conclusion of the experiments. The aim of this article was to determine whether animal experiments are scientifically or morally justified in bringing significant evidence in studies that may validate the influence of changes in the concentration of female hormones secreted by the ovaries in various phases of the menstrual cycle in young patients on the duration of an increased tooth movement rate in orthodontic treatment. Papers reporting the results of the original research into female hormones, either natural or exogeneous ones, likely to alternate the orthodontic tooth movement rate were critically evaluated in terms of animal selection. Thorough analysis supported by veterinary knowledge proved that none of the publications enabled an extrapolation of the results to humans. The evaluation of the relation between the rate of tooth movement upon loading with orthodontic forces and hormones either secreted during the menstrual cycle of women or released from the contraceptives already present in the market, does not require sacrificing laboratory animals.

## 1. The Use of Laboratory Animals in Orthodontics

Animal testing was and remains the only method of introducing a certain treatment and medical procedure on humans. The social acceptance of such experiments has been fervently debated since 13th century, when Saint Thomas Aquinas saw animals as useful machines, whilst Saint Francis of Assisi believed them to be humans’ smaller brothers. Descartes continued the debate in the 16th century. However, the real rebellion aimed at anti-vivisection was pioneered by a woman, Fanny Matin, who in 1845 married Claude Bernard who was infamous for conducting experiments on stray dogs [1]. Noteworthy, at that time women had no voting rights, therefore Fanny Matin’s opposition to vivisection reverberated across the scientific world and turned out to be the driving force behind the shift in the researchers’ approach to respecting animals. Female sensitivity was also the catalyst for a mass protest in London. A conflict between Professor William Bayliss, a scientist who experimented on a terrier dog, thus discovering hormones, and whom the majority of the society considered to be a sadist, ended with a bronze monument of a dog being erected in London. The fate of a simple terrier had become the spur for adopting legislation protecting laboratory animals. However, it was not until the 21st century that the world of science changed forever: animals now have their ‘protection’ under existing legislation, including Directive 2010/63/EU [2], and it is primarily recognized in European Union countries. This directive was transposed into Polish legislation in 2015 [3], and states that animals must be provided with medical and veterinary care and conditions adequate to their health and until their natural death regardless of whether the experiment has ended. However, it must be emphasized that animals subjected to experiments very rarely fully recover, so their “natural” death issue is rather controversial [4]. Furthermore, animals react and metabolize substances differently in comparison to humans. For example, the lethal dose of potassium cyanide for rabbits and mice is, respectively, twice and seven times higher (obviously relative to their body mass) than for humans. In this term, many researchers, such as Ober [5] as well as Azkona [6], believe that in the case of interdisciplinary research work, cooperation between clinicians and scientists and qualified veterinarians is necessary.

Since experimental animals are present throughout all fields of science related to human healthcare, unsurprisingly, they are also involved in the development of dentistry, specifically in the field of orthodontics. The treatment of malocclusions in the case of young adult women, aged 20 to 30, is naturally linked to a menstrual cycle, which constitutes a sequence of recurring fluctuations of hormones that prepare the body for potential pregnancy: estrogens and especially progesterone. These hormones regulate osteoblast activity and the production of collagen, responsible—among other things—for a proper periodontal structure [7,8,9,10,11,12]. The natural cycle of sex hormones physiological fluctuations is disrupted by ovariectomy or hormonal contraception taken by a patient. Although modern oral contraceptives contain much lower doses of hormones when compared to medication used in the past, they still may damage periodontium, as they contribute shifting a balance between aerobe and anaerobe microorganisms towards the latter [13,14,15].

Any impairment in the alveolar bone structure immediately affects the rate of orthodontically induced tooth movement. It is therefore quite likely that if loading the teeth is planned in accordance with the menstrual cycle, one obtains the most efficient tooth movement due to the highest “plasticity” of periodontal tissues, which results in shortening the treatment time. Nevertheless, the World Medical Association International Code of Medical Ethics (Helsinki Declaration, 1975, amended 2000) requires that medical support, including orthodontic procedures, be carried out on at least two species, animals, before it can be used in humans [16]. Therefore, since the orthodontic experiment must burden laboratory animals so much, the credibility of the results of hormonal and orthodontic tests obtained so far and their usefulness for the human population should be critically assessed. Especially that, according to statistical data, about 50% of laboratory animals are euthanized at the conclusion of the experiments [17,18].

In this commentary, we aimed to determine whether animal experiments are scientifically or morally justified in bringing significant evidence to studies that may validate the influence of changes in the concentration of female hormones secreted by the ovaries in various phases of the menstrual cycle in young patients on the duration of an increased tooth movement rate in orthodontic treatment.

To this end, we searched PubMed, Elsevier ScienceDirect Journals, and EBSCOhost (Medline) electronic databases for laboratory research performed on animals, concerning orthodontic movement depending on the level of hormones during the menstrual cycle and while using hormonal contraceptives. We included review, clinical, comparative, intervention papers, and unusual cases, using the following keywords: estrogen, progesterone, hormonal contraception, menstrual cycle, heat, orthodontics, and orthodontic treatment. We disregarded the research performed on humans, as well as in vitro or in silico texts. We found 12 studies published between 1997 and 2022, which discussed the related subjects we target in this review.

## 2. Selected Studies

The impact of female hormones and hormonal contraception on orthodontic movement has been the subject of research in various centers, done on different species of animals. Most tests were done on Wistar rats, i.e., white rats used in pharmacological, toxicological, nutrition, and behavioral testing, but similar studies were performed on rabbits and cats. The canines, premolars, and incisors were moved predominantly. Open orthodontic springs and nickel and titanium wires were usually used for this purpose, with some studies using bone anchoring, i.e., mini implants. Some of the animals had their blood tested, others had their vaginal mucus tested. The tooth movement ranges were measured on gypsum dental casts poured on the examination day or inside the animals’ mouths using a caliper. Most animals were euthanized after the research. Only healthy females took part in testing, with their own heat cycle or sterilized and exogenously administered with hormones [15,19,20,21,22,23,24,25,26,27,28,29].

The publications were divided into groups and specific publications were selected based on the scheme shown in Table 1.

## 3. Cross-Species Comparison with Humans

Factors crucial for hormonal and orthodontic experiments, differentiating women from female animals selected for testing, are shown in Table 2.

## 4. Rats

**Figure 1:**
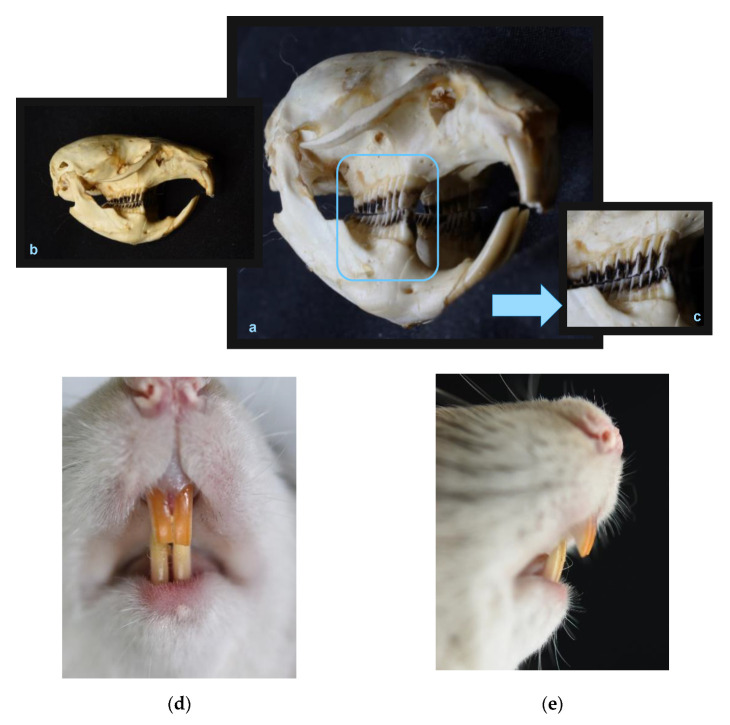
Rat teeth in a skull: semi profile view (**a**), lateral view (**b**), premolars and molars view (**c**). Rat teeth: incisors, frontal view—enlarged overbite (**d**) and incisors, lateral view—enlarged overjet (**e**) (by Małgorzata Peruga).

Rats (Figure 1a,b) procreate quickly and their genes have also been well-mapped. Their transgenic strains, possible to be created nearly exclusively in small rodents, regardless of certain difficulties in maintaining their permanence, are still an important aspect favoring the selection of rats as experimental animals. This choice is also determined by the size of the animal, which facilitates the conduct of treatments and the preparation of histopathological materials. Rats are also cheap, which helps in testing their large groups. This is likely why rats were experimental animals in the majority of reviewed studies [15,19,20,21,22,23,24,25,27,28]. The evaluated studies theoretically demonstrated that the forced tooth movement rates are dependent on menstrual cycle or hormonal contraception. However, studies by Guo et al. [19,20,22] and Zhao et al. [21,23], performed at various intervals on various groups of rats, do not provide data for the measurements of the tooth movement and orthodontic biomechanics in relation to the heat cycle. The full-text articles are available only in Chinese, so we were able to review their abstracts, which merely summarized the research and did not fully report the results. We attempted to contact the researchers, but they did not reply to our emails. In turn, Mackie et al. [28] performed their tests on a strain other than Wistar, namely Sprague Dawley characterized by longer jaws, which naturally increases the distance between the incisors and the molars. Longer wires and springs are more easily deformed during chewing, when the so-called trampoline effect caused by the occlusal forces becomes evident [31]. Furthermore, the study used very young, 6-week-old specimens additionally subjected to stress that might drastically change their hormone levels; rats are less willing to reproduce when experiencing distress. As many as 4 specimens out of 55 died from stress, which undermines the entire test results, as the intensity of the metabolism is significantly increased during stress. In another study, also done on young rats, Olyaee et al. [15] used a spring between two incisors, which also challenges the reliability of the outcomes; due to the high probability of the not-fused palatal suture, the diastema quite likely resulted not from the tooth movement itself, but from separating the maxillary halves. Thus, it may be concluded that choosing a rat as an animal model for hormonal and orthodontic research is also unfounded. Although, similarly to humans, gaps between rat teeth (Figure 1c–e) are sufficiently wide to secure the natural drift of molars, and the teeth may be affected by caries; this is where the similarities end. Aside from the many evident differences (Table 2), the incisors in rats have enamel only on their front surfaces and they grow throughout the animals’ lifetimes, which requires continuous sharpening of those teeth; in addition, the enamel is harder than metals such as iron, platinum, and copper. Continuous tooth eruption does not provide anchorage and sufficient control over the direction of force, which may lead to bias while interpreting the published data.

As for the periodontal ligaments (PDL) of rats, they are built of connective tissue collagen fibers, which requires vitamin C (synthesized by rats in their kidneys or liver) to grow. Rats therefore do not have to obtain vitamin C exogenously, in contrast to humans, who do not produce L-gulonolactone oxidase (GULO), an enzyme contributing to vitamin C synthesis. It is worth noting here that although rats without GULO have been bred, they did not correctly reproduce the vitamin deficits seen in humans, which made the extrapolation of the obtained results impossible.

## 5. Rabbits

**Figure 2:**
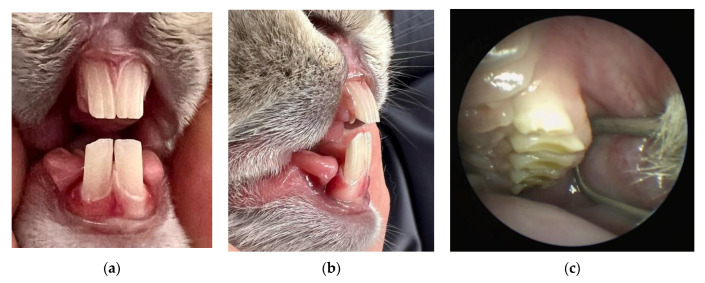
Rabbit teeth: incisors frontal view—overbite (**a**) incisors lateral view—overjet (**b**) and premolars and molars view (**c**) (by Jakub Kotowicz).

**Figure 3:**
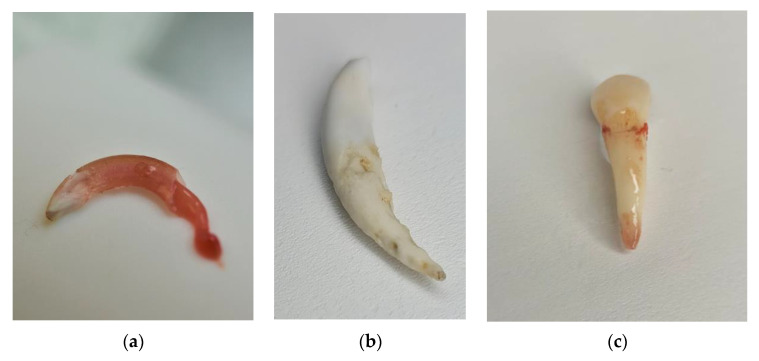
Rodent incisor with open top and living pulp (**a**). Comparison with thecodont and brachydont dog tooth (**b**) and human tooth (**c**) (by Małgorzata Peruga).

**Figure 4:**
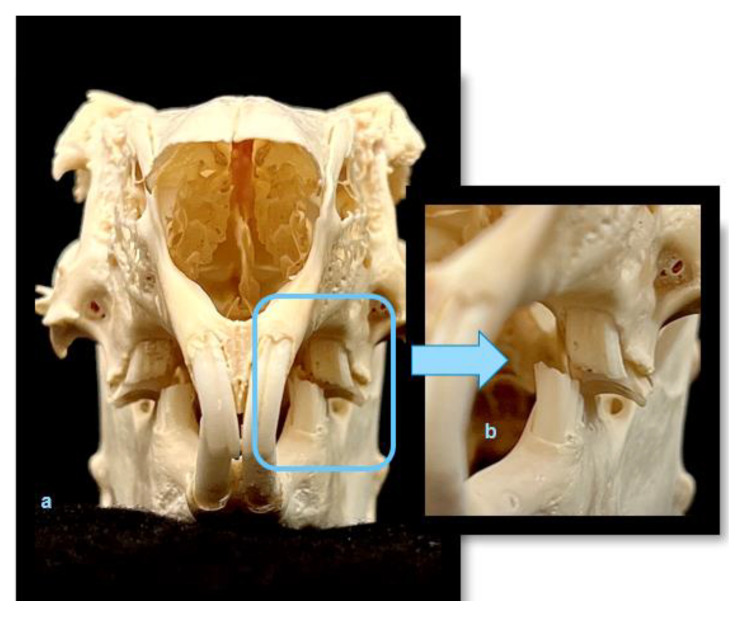
Anisognathism in rabbits: in 1:1 scale (**a**), enlarged (**b**) (by Małgorzata Peruga and Jakub Kotowicz).

Rabbits, and specifically their thigh bones, are used in dentistry as a material for research into the osseointegration of implants. However, rabbit is an animal with a fragile anatomical structure, in particular its limbs, which often fracture under a load. Rabbits not only show little aggression toward humans but are also the smallest and the cheapest animals whose sperm can be harvested and used for artificial insemination. They produce tears and have large eyeballs, which facilitates the testing of chemical substances. However, apart from research into irritating substances, there is scant information available on other experimental studies. Poosti et al. [29] demonstrated that orthodontic movement changed after a female rabbit was provided with hormones from a human female that have a different chemical structure to their own hormones. The teeth of rabbits (Figure 2a–c), namely the incisors and the molars, can grow throughout their lifetimes. The incisors grow even two to three millimeters per week. Rabbits’ teeth consist of clinical and anatomic crowns almost entirely covered by a layer of enamel absent at the top of the tooth, in the growth center. The PDL area is very limited (Figure 3a–c), which modifies the tooth behavior under loading with occlusal or orthodontic forces [32,33,34]. Mastication is also very different compared with humans. After reaching occlusal interdigitation, the rabbits’ mandibular teeth rest on pegs (second part of the maxillary incisors) in a reversed overjet. To achieve normal occlusion, a rabbit must unilaterally and partially dislocate the mandibular condylar process from the fossa to close the arcade (Figure 4a,b).

Choosing rats and rabbits as test animals to assess the tooth movement during either a menstrual cycle or the administration of hormonal contraceptives is also controversial due to the fact that the estrus of these animals is too short [15,19,20,21,22,23,24,25,27,28,29,32,33,34,35] (Table 2) to notice visible changes in the three-stage process caused by orthodontic forces. Moreover, small animals do not match human biologically and do not live for long, not allowing for longitudinal studies.

## 6. Cats

**Figure 5:**
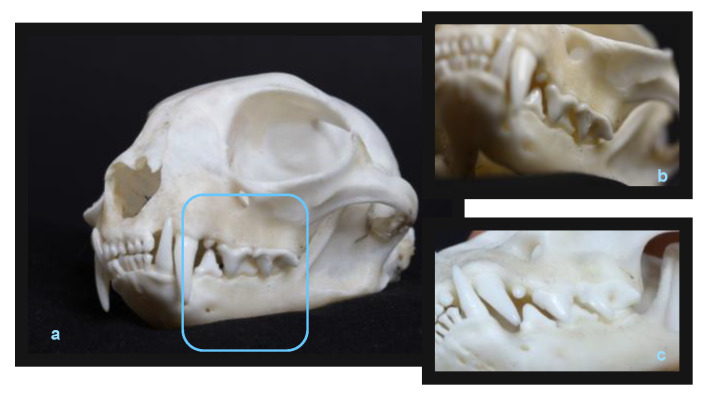
Cat skull and teeth: semi profile view (**a**), lack of adhesion of the side teeth—scissor arrangement (**b**,**c**) (by Małgorzata Peruga).

Celebi et al. [26] used the domestic cat as a research model in hormonal testing. However, cats have no masticating surfaces (Figure 5a–c). The arrangement of their teeth in the dental arches and high degree of nodularity precludes using cats for orthodontic research, unless the bite is raised, which the authors did not mention describing their methodology (Table 2). Although cats are used as laboratory animals, due to the fact that their brain demonstrates the closest similarity to humans, these animals are primarily used in neurological, ophthalmological, and immune deficiency research, and not in hormonal and orthodontic studies.

In a nutshell, a good understanding of the anatomy and physiology of experimental animals will provide us with information that animals such as rats, rabbits, or cats were lacking validity as laboratory animals for orthodontic movement research. The analyzed articles (Table 1) found numerous errors in the research assumptions, due to the fact that the authors did not take into account the important aspects that were pointed out, such as the different anatomical structure of teeth and periodontal and completely different occlusions.

## 7. Hormone Cycle

Most studies into the relation between the rate of the tooth movement and the action of hormones secreted during the menstrual cycle were done on animals, who were administered human progesterone, estrogens, and relaxin [36,37,38,39,40,41]. As far as progesterone is concerned, experimental research on rats has demonstrated that the hormone modified the orthodontic movement of their teeth by affecting the periodontium and elasticity of the cortical plate of the alveolar process. On the other hand, the long-term administration of progesterone to rabbits resulted in a reduced rate of the tooth movement; the authors concluded that it was due to the fact that osteoclasts are observed primarily 2 days after an orthodontic force is applied [31]. Administering relaxin to rats resulted in an increase in the rate of orthodontic tooth movement when compared to control groups, as well as the stretching of periodontium made of soft tissue [42,43,44,45]. Unfortunately, as the normal hormonal cycle of animals was disrupted in every reviewed study, it cannot be stated with certainty whether the changes in the rate of tooth movements were caused only by the excessive amount of artificially introduced hormones or a disruption in the natural hormonal balance, particularly given the short observation period.

## 8. Orthodontic Materials

Most of the reviewed studies used materials made from nickel and titanium (NiTi) alloy, which has been popular in orthodontic treatment since the 1970s. Its elasticity modulus approximately equals 20% of the modulus of the stainless steel, which secures a very wide scope of working elasticity. The complex metallurgical nature of nickel and titanium materials and its relation to clinical application have been the subject of many scientific studies. It has been demonstrated that the NiTi alloy has two phases. The first phase, austenitic, has an ordered structure, whereas the second phase, martensitic, is a highly strained body-centered tetragonal form. Shape memory is linked to the reversible transformation of martensite into austenite, which occurs as a result of a crystallographic process. The microstructure of alloys in the temperature found in the human oral cavity (36.1–37 °C) is not fully austenitic. The temperature of the complete transformation to austenite is 40 °C, which is evidently higher than the temperature in the oral cavity. That is why the alloy will behave differently in patients breathing through their mouth (27 °C) or consuming hot food (40 °C). The issue of the natural bodily temperature of animals [46] cannot therefore be ignored, as it changes the behavior of the nickel and titanium spring.

## 9. Conclusions

Many experimental tests have so far been done on various species of animals to obtain a better understanding of their biological reactions to orthodontic forces. Unfortunately, one of the main problems related to animal experiments is the fact that their results cannot be extrapolated to humans. The two- or even four-year lifespans of rats and rabbits, respectively, together with short estrus, enable us only to observe the immediate effects of the tooth loading, which are impossible to extrapolate on humans due to severe dissimilarities of the tooth anatomy, their PDL, as well as female hormone secretion cycles. Additionally, the lack of long-term results, which are the most reliable scientifically, is a serious limitation of the so-far designed experimental studies. The orthodontic treatment of humans with fixed appliances has been ethically accepted since at least the beginning of the previous century. Thus, evaluating the relationship between the rate of tooth movement upon loading with orthodontic forces, and hormones either secreted during the menstrual cycle of women or released from the contraceptives already present in the market, does not require experimental testing. It is enough to interview the patient and to determine the adequate timing of a force application, with subsequent measuring of the rate of the tooth movement during scheduled appointments. Perhaps a “reductio ad absurdum” argument is irresponsible; however, orthodontic treatment is carried out successfully and, above all, does not involve any risk, because the general principles of mechanics and biomechanics are known. Our results should encourage researchers to analyze the methods and selection of animals for research in more detail. There is no moral justification for performing orthodontic examinations on rats, rabbits, or cats, which we have proved.

## Figures and Tables

**Table 1:** List of research methods and results obtained in papers analyzed as part of the review.

Authors	Animals and Their Division	Tooth and Type of Tooth Shift	Material Used	Method Used to Test the Hormone Level	Results
Olyaee et al. [15]	Wistar rats Age—3 months N = 48 m = 250 ± 25 g females	Central incisors tipped distally	SS spring with a diameter of 0.35 mm	Administration of ethinyl estradiol/norgestrel	Ethinyl estradiol/norgestrel (oral contraceptives) can decrease the amount of tooth movement
Guo, Zhao, Chen [19]	Wistar rats Age—not given N = 120 m = not given females	Not given	Not given	Estradiol level in serum and periodontium tissue using radioimmune and immune-cytochemical methods	Estrogen affects teeth movement
Zhao, Than, Guo, Chen [20]	Wistar rats Age—not given N = not given m = not given females	Not given	Not given	Estradiol level in serum and periodontium tissue using radioimmune and immune-cytochemical methods	Estrogen affects teeth movement
Guo, Zhao, Chen [21]	Wistar rats Age—3 months N = 80 m = not given females	Left upper incisor and left upper molar Tipped	Not given	Not given	Teeth movement dependent on cycle
Zhao, Than, Guo, Chen [22]	Wistar rats Age—not given N = not given m = not given females	Not given	Not given	Not given	Estrogen affects teeth movement
Guo, Zhao, Chen [23]	Rats, strain not given Age—not given Not given N = 200 m = not given females	Not given	Not given	Estradiol level in serum	Estrogen affects teeth movement
Haruyama et al. [24]	Wistar rats Age—10 weeks N = 85 m = 136 g females	right and left, first upper molar Tipped	NiTi spring with a diameter of 0.012 inch	Acc. to estrous cycle with vaginal smear	Estrus can increase tooth movement
Tan et al. [25]	Wistar rats Age—3 months N = 200 m = 300 g females	Left distal incisor and first left molar Tipped	NiTi spring with a diameter of 0.012 inch	Monitoring estrus cycle and vaginal smear	Estrus can increase tooth movement
Celebi [26]	Domestic cat Age—2–4 years N = 18 m = not given females	Jaw canine and mini implant Tipped	NiTi spring with a diameter of 0.2 inch	According to estrus cycle	Teeth movement speed was higher in sterilized specimens
Sirisoontorn [27]	Wistar rats Age—10 weeks N = 10 m = 170–190 g females	Left distal incisor and and first left molar Tipped	NiTi spring with a diameter of 0.012 inch	Monitoring estrus cycle and vaginal smear	Teeth movement speed was higher in sterilized specimens
Mackie et al. [28]	Sprague Dawley rats Age—6 weeks N = 55 m = 160 ± 20 g females	Right distal incisor and first right molar Tipped	NiTi spring with a diameter of 0.03 × 0.01 inch	Not given	Estrus can increase tooth movement
Poosti et al. [29]	Rabbit Age—8 weeks N = 24 m = 1850 g females	Central incisors tipped distally	SS spring with a diameter of 0.014 inch	Administration of progesterone	Progesterone affects tooth movement

Legend: N—number of animals used in testing; m—body mass of animal used in testing; SS—stainless steel; NiTi—nitinol.

**Table 2:** Comparison of laboratory animals in terms of dentition, periodontium, reproductive cycle, body temperature.

Species	Size	Dental Formula	Teeth	Periodontium	Hormones	Body Temperature
Human (*Homo sapiens*)	Target species	$\frac{212}{212}$ $\frac{2123}{2123}$	Diphyodont Heterodont Bunodont	Thecodont Brachydont	Cycle 24–33 days Ovulation 24 h	36.6 °C
Laboratory rat (*Rattus*)	Acceptable size	$X\;\frac{1003}{1003}$	Monophyodont heterodont	Thecodont Monophyodont Incisors—hypsodont, elodont Molars—brachydont	Cycle 4–5 days Spontaneous ovulation Estrus 10–20 h	37.5–39 °C
Rabbit (*Oryctolagus cuniculus*)	Acceptable size	$X\;\frac{2033}{1032}$	Monophyodont Heterodont	Thecodont Hypsodont Elodont	Cycle 16 days Induced ovulation	38.5–40 °C
Domestic cat (*Felis catus*)	Acceptable size	$\frac{313}{312}$ $\frac{3131}{3121}$	Diphiodont Heterodont Secodont	Thecodont Brachydont	14–21 days	38–39 °C

Definition of terms (acc. to Kobryń, H.; Kobryńczuk, F.; Krysiak, K. *Anatomia Zwierząt Tom 1–3 (Animal Anatomy Vol. 1–3)*; PWN: Warsaw, Poland, 2011 [30]): X- does not occur; monophyodont—having one set of teeth; diphyodont—having two set of teeth; heterodont—with tooth shape differences between incisors, canines, premolars, and molars; thecodont—tooth embedded in a socket; brachydont—having short crowns with short growth time; hypsodont—having high crowns with long growth time; secodont—having sharp enameled teeth; bunodont—having rounded enameled teeth; elodont—teeth with an open top. An animal’s dentition for either deciduous (first fraction) or permanent (second fraction) teeth expressed as a dental formula, written in the form of a fraction, as $\frac{I.C.P.M}{I.C.P.M}$ maxillary arch (above the line) and mandibular arch (below the line) I—incisors, C—canine, P—premolar, and M—molar, f.ex human 2123—2-I, 1-C, 2-C, 3-M.

## Data Availability

Not applicable.
